# Applying LIBS, SEM/EDX, and FTIR spectroscopic analysis for the conservation of cairene architectural heritage

**DOI:** 10.1038/s41598-026-56422-8

**Published:** 2026-06-09

**Authors:** Raghda Hosny El-Saeid, Anwer Mahran, Mona F. Ali, Mohamed Abdel-Harith

**Affiliations:** 1 National Institute of Laser Enhanced Science, Cairo University, Giza, 12613 Egypt; 2 Conservation Department, Higher Institute of Tourism and Restoration, Alexandria, Egypt; 3 Faculty of Archaeology, Cairo University, Giza, Egypt

**Keywords:** Al-Qazdughli Palace, Wall paintings, Laser-induced breakdown spectroscopy (LIBS), SEM/EDX analysis, FTIR spectroscopy, Cultural heritage conservation, Chemistry, Environmental sciences, Materials science

## Abstract

This study investigates the wall paintings of Al-Qazdughli Palace, an early twentieth-century landmark in Cairo’s historic Garden City district near Simon Bolivar Square, using a multi-analytical spectrochemical approach. As an important example of Cairo’s architectural heritage, the palace has experienced extended neglect and significant deterioration. Laser-Induced Breakdown Spectroscopy (LIBS) using an Nd: YAG laser at 1064 nm was used to examine six pigment colors: gold, brown, light green, blue, bright red, and dark red. The gold pigment showed strong signals for Zn, Ag, Ba, Au, Cu, and Pb; Zn was dominant in the brown pigment; and the blue pigment contained Ti, Cu, and Zn. The light green pigment revealed Ca, Cu, Zn, and Pb; the bright red pigment showed Ca, Fe, Cr, Zn, Hg, and Pb; and the dark red pigment contained Ti, Mn, Fe, and Zn. The LIBS findings were confirmed through SEM/EDX analysis, which produced consistent elemental results. Optical microscopy enabled non-invasive examination of paint-layer surface morphology and microstratigraphy. FTIR analysis of selected pigments also identified carbonyl ester bands (1750–1740 cm⁻^1^), indicating oil-based binding media. Together, these results highlight LIBS, supported by complementary techniques, as an effective quasi-non-destructive tool for the in situ analysis of wall paintings and for informing conservation and restoration strategies.

**Supplementary Information:**

The online version contains supplementary material available at 10.1038/s41598-026-56422-8.

## Introduction

Al-Qazdughli Palace, situated in Cairo’s Garden City district near Simon Bolivar Square, is a distinguished example of European-inspired architecture in Egypt during the early twentieth century, a period marked by British protectorate rule^1^. The Soares family initially owned the building before being acquired by Emmanuel Qazdughli, a Greek entrepreneur who settled in Egypt during the reign of Khedive Ismail. In recognition of his legacy, the palace was named after him. The Qazdughli family, of Ottoman or Circassian origin, held prominent social positions during the Alawite dynasty and settled on the estate. The palace was designed by the Austrian architect Eduard Matzke, who is known for several notable buildings in Cairo, including the Austro‑Hungarian Rudolf Hospital, the main synagogue, and the Crédit Foncier bank. Al‑Qazdughli Palace thus reflects both the cosmopolitan character of Cairo at the turn of the twentieth century and the interplay between European architectural trends and local Egyptian traditions.

Despite its historical and artistic significance, the palace has suffered decades of neglect and insufficient maintenance. Following the 1952 Revolution, it was converted into a public school, which accelerated its physical deterioration. The building was further vandalized, looted, and partially burned during the 2011 riots and has remained abandoned since 2008^2^. Although in poor condition, Al‑Qazdughli Palace still stands as a remarkable landmark of Cairo’s architectural heritage, illustrating the long‑standing dialogue between European and Egyptian styles.

In response to its critical state, a governmental restoration project has been initiated to conserve the palace, including its richly decorated wall paintings. Effective conservation of such paintings requires a detailed understanding of the materials used, particularly the pigments and binding media. Determining the precise elemental and molecular composition of these materials not only guides the selection of appropriate conservation treatments but also sheds light on the technological choices and artistic practices of the late nineteenth and early twentieth centuries.

A comprehensive characterization of architectural heritage typically relies on a combination of advanced scientific techniques. By examining the chemical and structural features of pigments and binders, scholars can infer provenance, manufacturing methods, and technological relationships with other contemporary or historical works. Among the most widely used analytical tools in archaeological and heritage science are X‑ray and ion‑beam‑based methods, such as scanning electron microscopy with energy‑dispersive X‑ray spectroscopy (SEM‑EDX)^3,4^, X‑ray fluorescence spectrometry (XRF)^5,6^, and proton‑induced X‑ray emission (PIXE)^7,8^. These non‑destructive or minimally invasive techniques enable detailed elemental analysis while preserving the integrity of valuable artifacts.

In recent decades, significant progress has also been made in the application of spectroscopic methods to archaeological and historical materials. Raman spectroscopy^9^, Fourier‑transform infrared spectroscopy (FTIR)^10,11^, and laser ablation inductively coupled plasma mass spectrometry (LA‑ICP‑MS)^12,13^ have become essential for elucidating the physicochemical properties of ancient objects. These techniques provide high‑resolution spectrochemical information that can reveal material composition, production technologies, and degradation processes. However, their broader routine application may be constrained by factors such as the need for sample preparation, relatively long analysis times, and high operational costs.

Over the past two decades, laser‑induced breakdown spectroscopy (LIBS) has emerged as a powerful complementary technique for the study of archaeological and historical artifacts, combining rapid data acquisition with quasi‑non‑destructive sampling. LIBS operates by focusing a high‑energy laser pulse, typically from an Nd: YAG laser, onto the surface of a sample to generate a transient microplasma^14–16^. The emission from this plasma plume is spectrally resolved and analyzed to determine the elemental composition of the material. A standard LIBS setup consists of a laser source, focusing optics, a light‑collection system, and a spectrometer equipped with a suitable detector for dispersed spectral‑line detection. This configuration provides both qualitative and semi‑quantitative elemental information. It is particularly advantageous for in situ measurements in museums or on archaeological sites, where instrument portability, minimal sampling, and little or no sample preparation are highly desirable.

In the present work, a multi‑analytical approach is applied to the wall paintings of Al‑Qazdughli Palace. Digital optical microscopy (OM), scanning electron microscopy (SEM) equipped with energy‑dispersive X‑ray spectroscopy (EDX), Fourier‑transform infrared spectroscopy (FTIR), and Laser‑Induced Breakdown Spectroscopy (LIBS) are combined to obtain a comprehensive characterization of the pigments and binders. Microscopic observation is essential for identifying the sequence and stratigraphy of the paint layers, as well as their morphology, color, and texture. SEM‑EDX provides detailed information on the elemental composition and microstructure of pictorial layers and has been widely used for the characterization of artworks and archaeological objects. FTIR offers insights into the molecular structure and bonding environment of both organic and inorganic components^17^, enabling the identification of binding media and degradation products. LIBS data are cross‑validated against EDX measurements to ensure the reliability of the elemental assignments.

The results of this integrated investigation shed light on the materials and painting technologies employed in Egypt during the transition from the late nineteenth to the early twentieth century. Moreover, the analytical evidence obtained in this study forms a scientific basis for developing a targeted conservation and restoration plan for the wall paintings of Al‑Qazdughli Palace. It contributes more broadly to the understanding and preservation of Cairo’s modern architectural heritage.

## Materials and methods

### Samples and site description

**Fig. 1:**
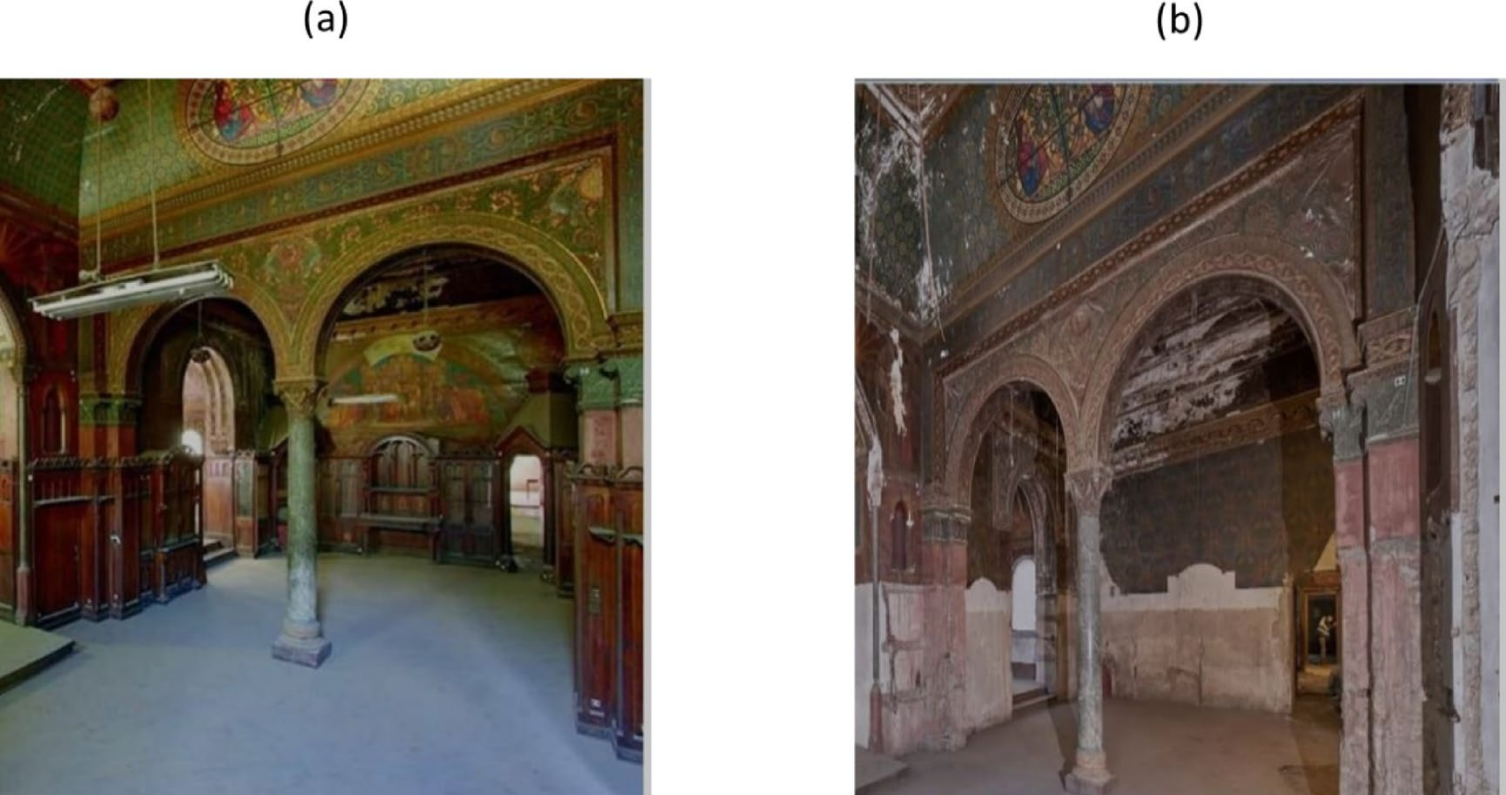
Site overview of Al-Qazdughli Palace: (**a**) the main lobby and its original historical decorations; (**b**) view of the deteriorated and damaged interior sections showing significant loss of some paint layers.

The samples investigated in this study were retrieved from the wall paintings of Al-Qazdughli Palace, located in Cairo’s historic Garden City district. Six distinct pigment colors were selected for analysis: golden, brown, light green, blue, bright red, and dark red. Figure 1 illustrates an interior view of the wall paintings of Al-Qazdughli Palace before and after restoration: (a) showing the original decorative architectural painting; (b) the present condition of the deteriorated walls, with faded paintings and structural neglect after exposure to the burning.

### Laser-induced breakdown spectroscopy (LIBS)

The LIBS experimental configuration followed the previously described setup^18,19^. A Q-switched Nd: YAG laser (Brio, Quantel, France) operating at its fundamental wavelength of 1064 nm was used as the excitation source. The laser delivered 5 ns pulses at 20 Hz, with a pulse energy of 45 mJ, as measured with a SCIENTECH AC5001 (Boulder, CO, USA) Joule meter. The beam was focused onto the sample surface using a 5 cm focal-length quartz plano-convex lens, creating a spot size of approximately 26 µm. Samples were positioned on a high-precision XYZ micrometric translation stage to maintain an optimal working distance. The resulting plasma emission was collected using a 600 µm core-diameter fused silica optical fiber and analyzed using an echelle spectrometer (Mechelle 7500, Multichannel, Sweden) coupled with an intensified CCD camera (DiCAM-Pro, PCO Computer Optics, Germany).

To optimize the signal-to-noise ratio and minimize electronic background interference, the ICCD was operated with a delay time of 1.5 µs and a gate width of 2.5 µs. For each pigment, 50 single-shot spectra were recorded at different surface locations to prevent cumulative “crater effects” and ensure statistical representativeness. Data processing was managed via GRAMS/32 software (National Instruments, USA), while elemental line identification was performed using LIBS^++^ software^20^.

### Digital optical microscopy

Surface morphology and microstratigraphic features on exposed painting edges were examined non-invasively using a portable digital microscope, equipped with a high-speed DSP and a high-definition CMOS sensor. The system facilitated real-time surface analysis at magnifications up to 500x. This non-invasive step was critical for documenting the pigments’ physical conditions, identifying signs of organic or inorganic degradation, and guiding subsequent micro-analytical measurements.

### Scanning electron microscopy (SEM/EDX)

To validate the LIBS elemental findings, surface microstructure and semi-quantitative elemental compositions were analyzed using a Field-Emission Gun Scanning Electron Microscope (FEG-SEM; Quanta 250, FEI, Czech Republic). The microscope was integrated with an Energy-Dispersive X-ray Spectrometer (EDX; Oxford Instruments X-MaxN 80 mm^2^ detector).

Analyses were conducted under high vacuum (10^–6^ mbar) using an accelerating voltage of 15 kV, a beam current of 1 nA, and a working distance of 10 mm. The system provided secondary electron (SE) imaging resolution of 1.2 nm and an EDX detection limit of approximately 0.1 wt %.

### Fourier-transform infrared spectroscopy (FTIR)

The binding media utilized in the paintings were identified using a Jasco 6100 FTIR spectrometer (Tokyo, Japan). Spectra were acquired in transmission mode using a triglycine sulfate (TGS) detector. Data were collected in the mid-infrared region 4000–400 cm^−1^ with a resolution of 4 cm^−1^ and a scanning speed of 2 mm/s. This analysis was performed at the Projects Sector laboratories of the Egyptian Ministry of Tourism and Antiquities to characterize the functional groups of the organic binders.

## Results and Discussion

### Elemental Characterization of Pigments by LIBS

**Fig. 2:**
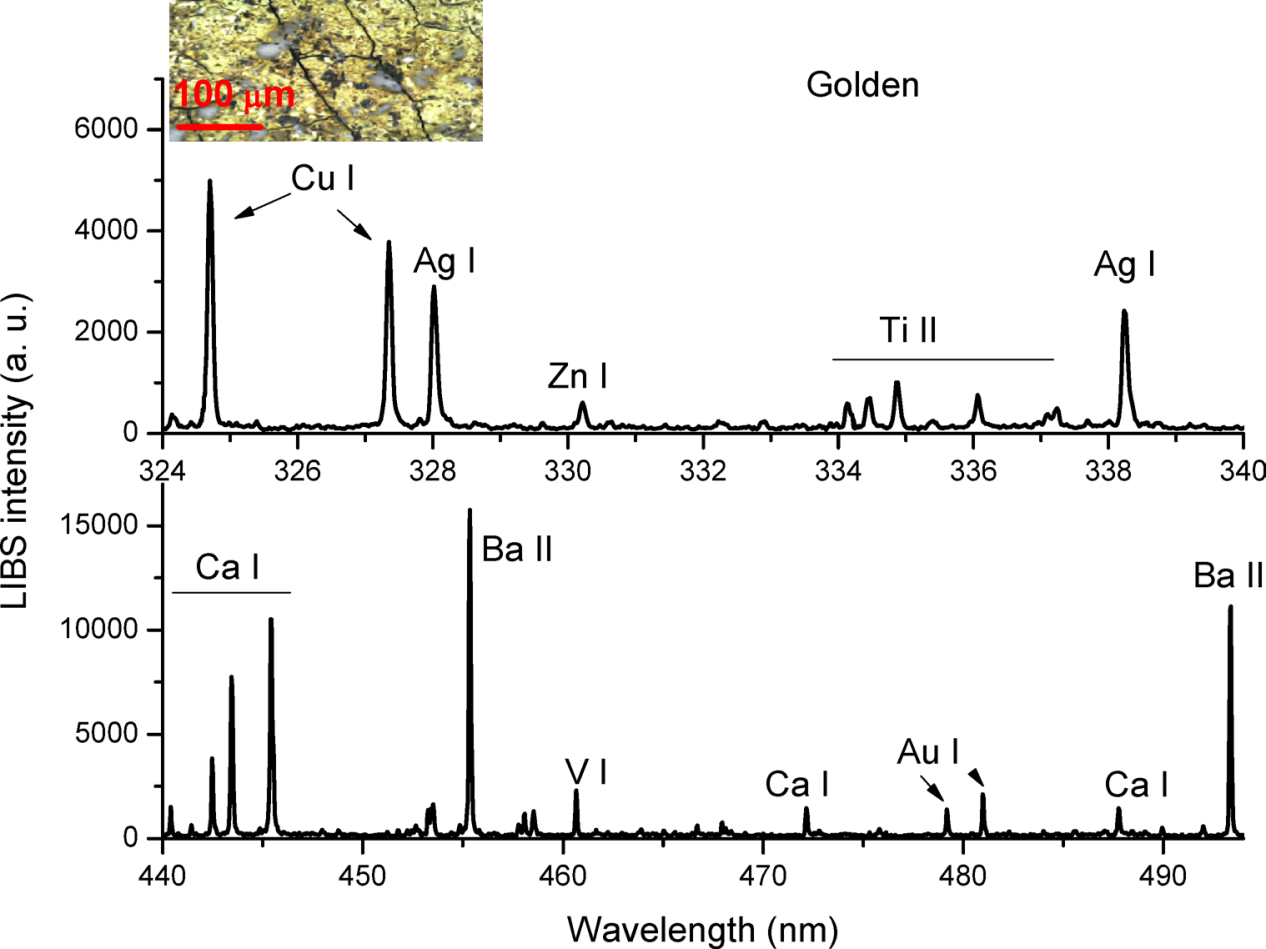
LIBS spectra of the Golden sample, highlighting characteristic gilding alloy lines (Au I, Cu I, Ag I) and background/preparation elements (Zn I, Ba II, Ca I). Inset: Surface optical micrograph showing the texture of the golden surface (scale bar: 100 µm).

**Fig. 3:**
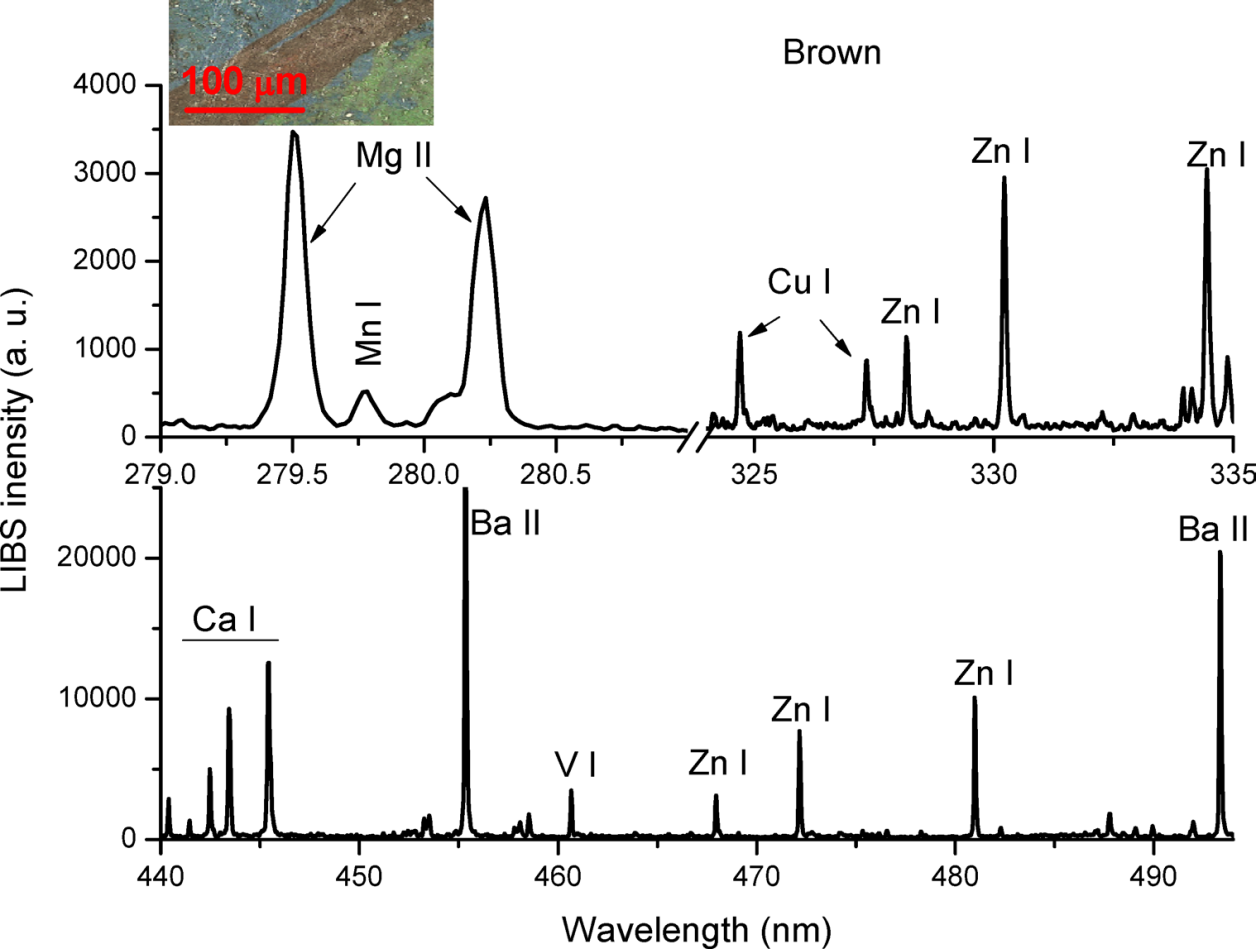
LIBS spectra of the Brown sample are dominated by zinc white ground matrix lines (Zn I, Ba II) alongside characteristic brown earth pigment markers (Mn I, Fe I, Mg II). Inset: Surface optical micrograph of the brown paint (scale bar: 100 µm).

**Fig. 4:**
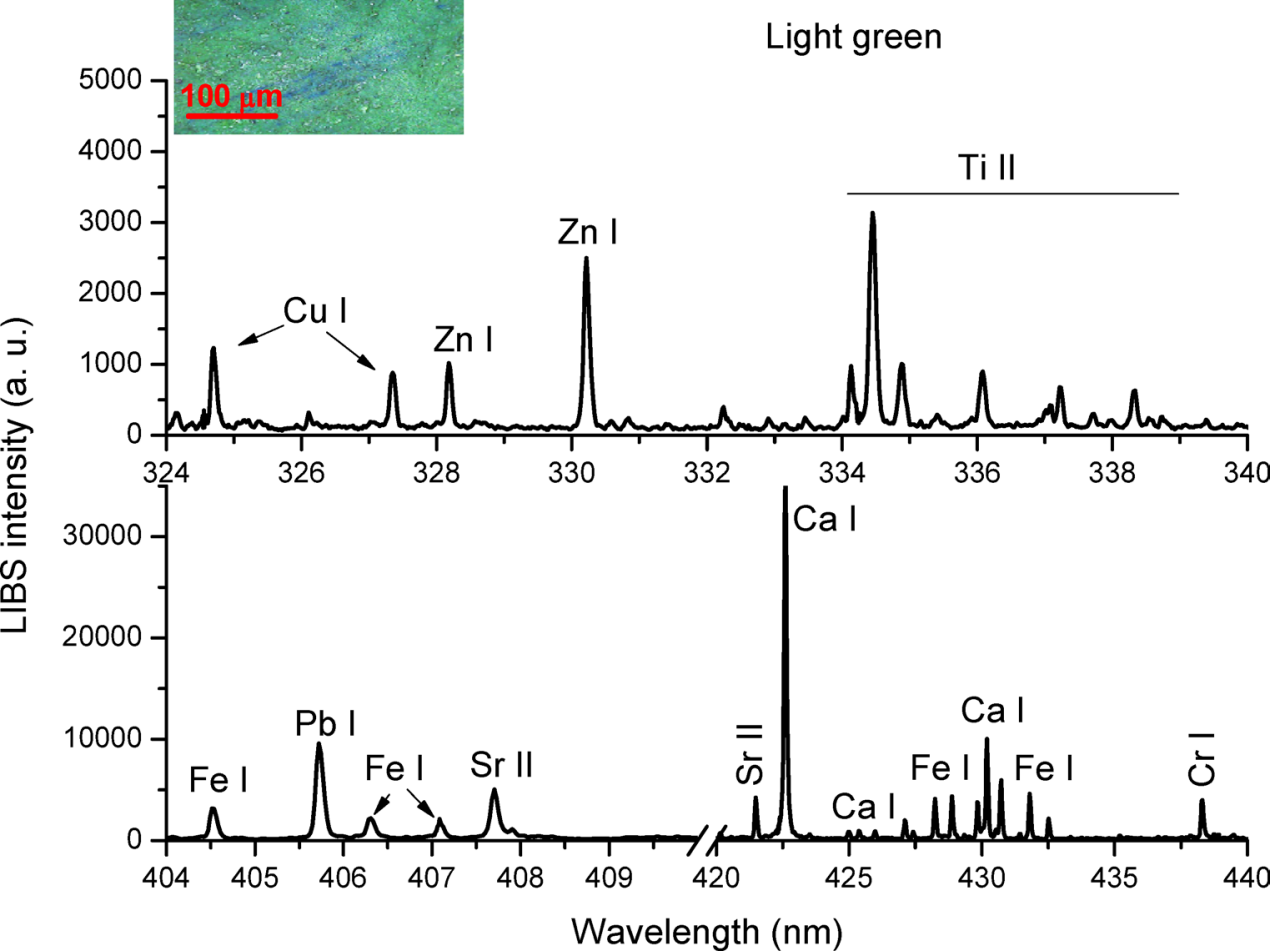
LIBS emission spectra of the Light green sample indicating synthetic chrome-based pigment signals (Cr I) and copper lines (Cu I) with ground elements (Ca I, Zn I, Ti I, Pb I). Inset: Surface optical micrograph of the light green layer (scale bar: 100 µm).

**Fig. 5:**
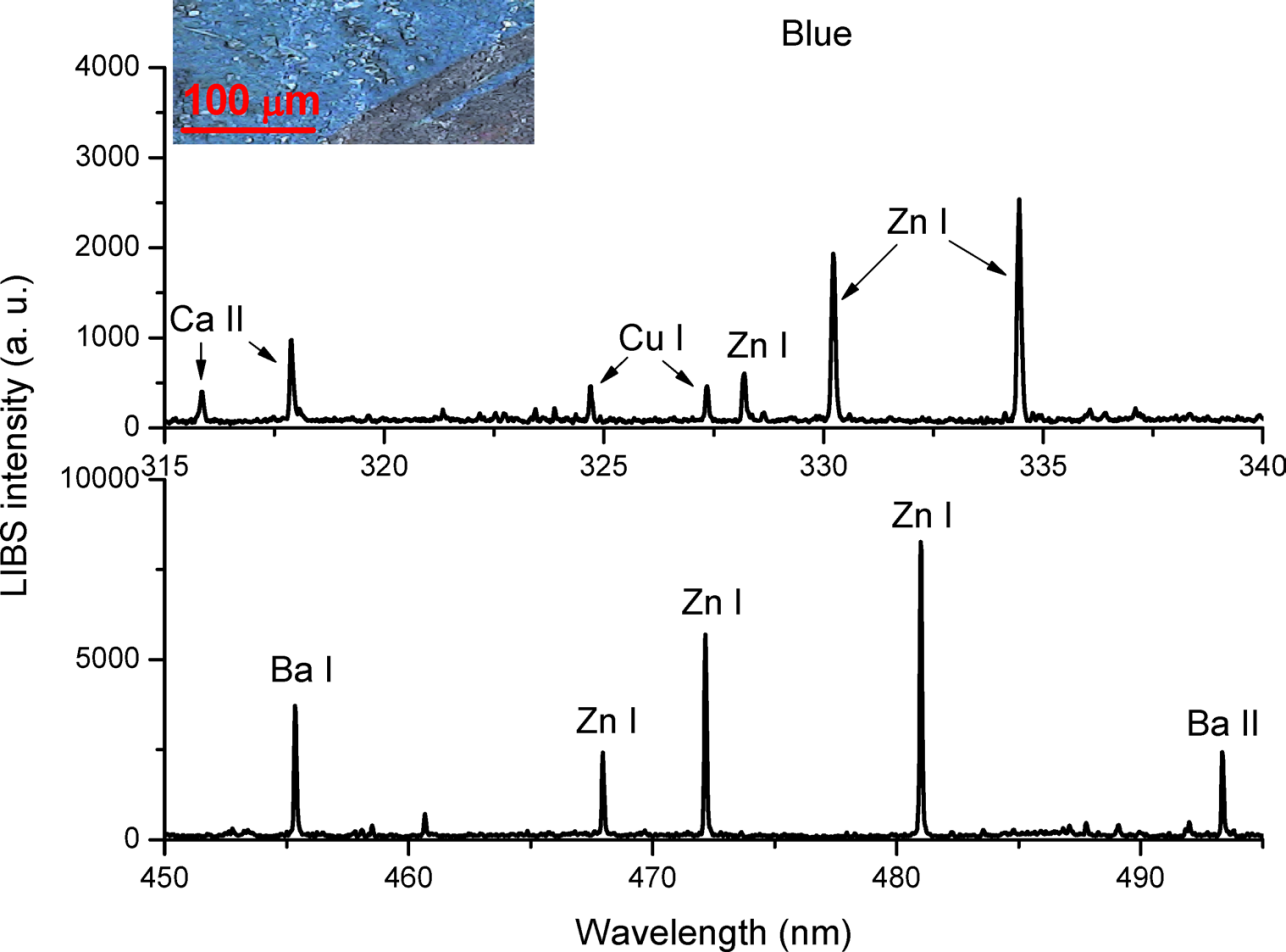
LIBS spectra of the Blue sample demonstrating diagnostic copper (Cu I) lines overlaid with high-intensity base matrix signals (Zn I, Ba II, Ca II). Inset: Surface optical micrograph of the copper-blue paint layer (scale bar: 100 µm).

**Fig. 6:**
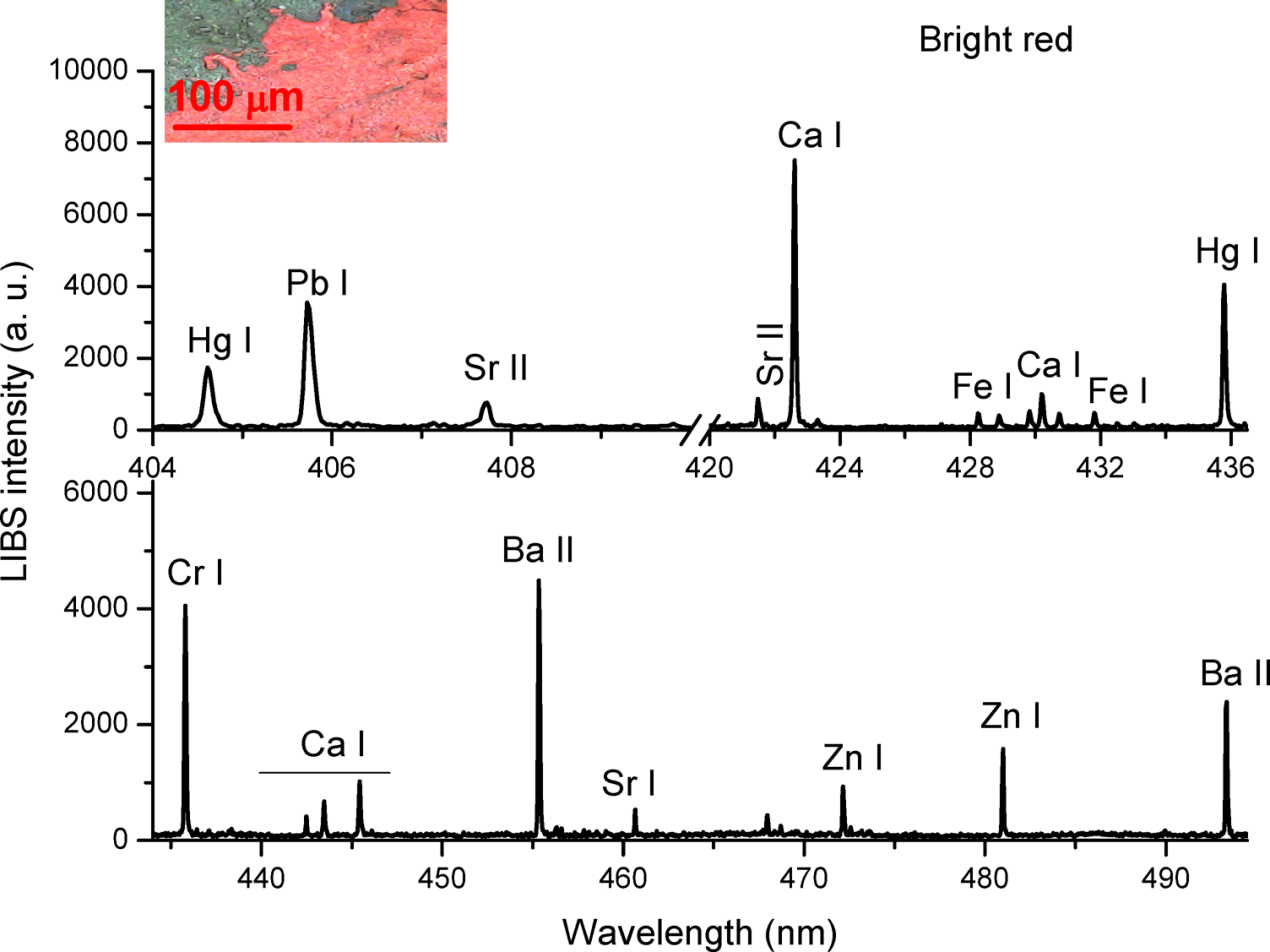
LIBS emission spectra of the Bright red sample clearly distinguish mercury (Hg) and lead (Pb) lines, indicative of a cinnabar mixed with a lead-based material. Inset: Surface optical micrograph of the brilliant red area (scale bar: 100 µm).

**Fig. 7:**
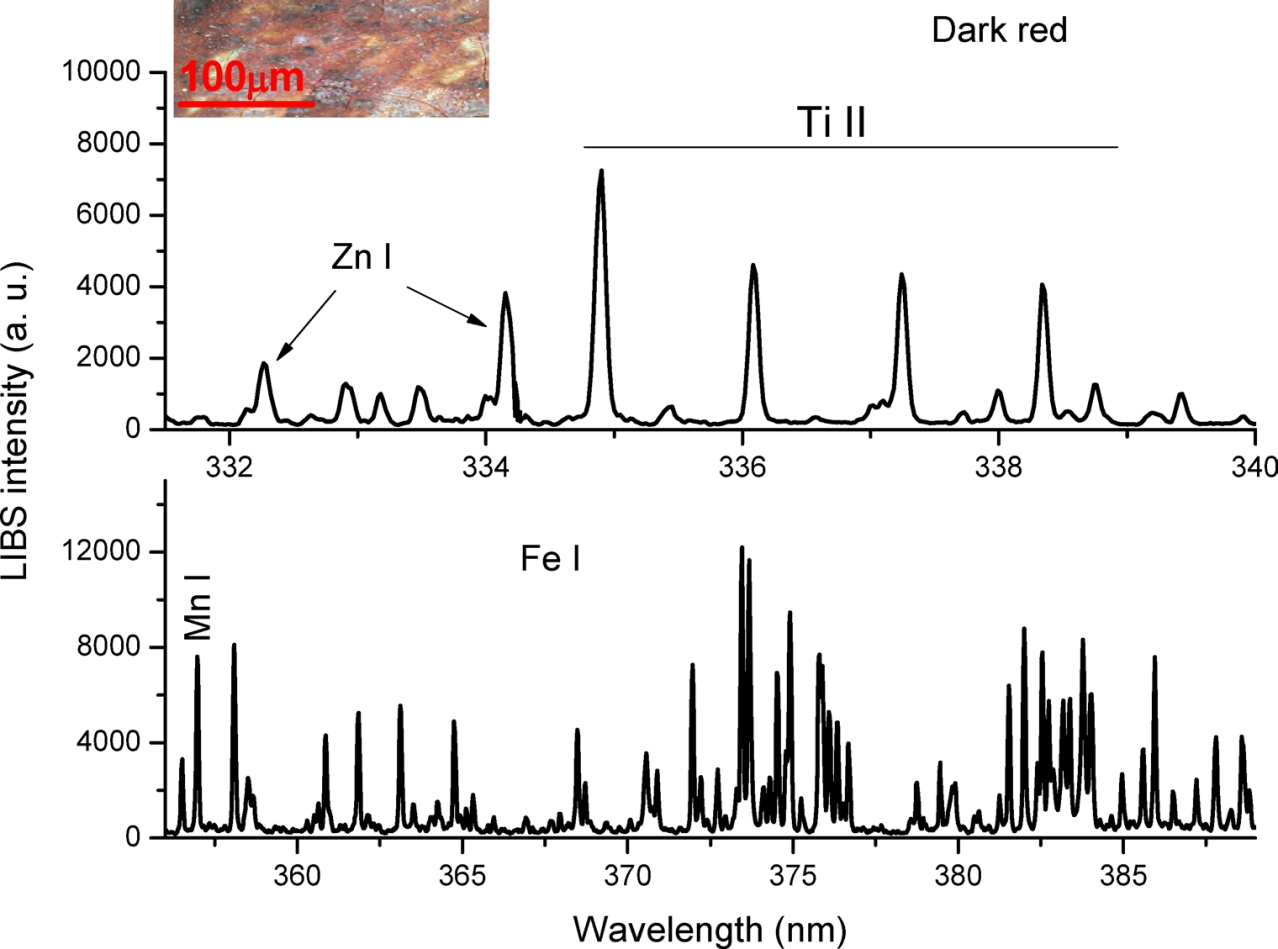
LIBS emission spectra of the Dark red sample exhibiting prominent iron (Fe) and manganese (Mn) lines characteristic of dark earth ochre, with smaller inputs of (Ti) and (Zn). Inset: Surface optical micrograph of the sample (scale bar: 100 µm).

Laser-Induced Breakdown Spectroscopy (LIBS) was used to identify the principal elemental composition of six distinct pigments from the wall paintings of Al-Qazdughli Palace. The LIBS spectra, presented in Figs. 2, 3, 4, 5, 6 and 7, reveal complex elemental fingerprints for each color. Elements such as Ca, Ti, Fe, Cu, and Zn appear across multiple samples but with varying relative intensities, reflecting differences in pigment formulation and preparation. This variability highlights the importance of comparative analysis between colors.

The golden pigment (Fig. 2) exhibits strong emission lines from Au, Cu, Ag, and Zn. The clear presence of gold (Au) lines, together with silver (Ag) and copper (Cu), common alloying elements in historical gold leaf, strongly supports the use of gold foil for gilding^21^. The signals of zinc (Zn) and barium (Ba) may originate from an underlying preparation layer or a mordant. In the brown pigment (Fig. 3), the spectrum is dominated by Zn, with significant lines for Mn, Fe, Cu, and Ba. The combination of manganese (Mn) and iron (Fe) is characteristic of umber or related earth pigments, which are naturally occurring mixtures of iron and manganese oxides that yield brown hues^22^. The high zinc content again suggests the presence of zinc-based white, either as an extender within the pigment or as part of the ground layer.

The light green pigment (Fig. 4) shows well-resolved lines for Cu and Zn, as well as for Pb, Fe, Ca, and Cr. The prominent copper lines are indicative of a copper-based green pigment, such as malachite or verdigris, which is often used in combination with other compounds^23,24^. The detection of chromium (Cr) is particularly noteworthy, as it suggests the possible use of a manufactured pigment, such as viridian, a hypothesis further examined by FTIR analysis. The blue pigment (Fig. 5) exhibits marked emission lines for Cu, Zn, Ca, and Si. The presence of copper is consistent with traditional blue pigments like azurite or Egyptian blue. The significant zinc and calcium signals likely correspond to a white base layer (e.g., zinc white, chalk) onto which the blue pigment was applied.

Analysis of the bright red pigment (Fig. 6) reveals a distinct elemental assemblage including Hg, Pb, S, Fe, and Cr. The presence of mercury (Hg) is a definitive marker of the brilliant red pigment cinnabar (HgS)^21^. The lead (Pb) signal may indicate the presence of a lead-based drier or a lead white pigment admixed with cinnabar to adjust hue or handling properties. The dark red pigment (Fig. 7) is dominated by strong signals from Fe and Mn, with smaller contributions from Ti and Zn. The predominance of iron and manganese oxides is typical of red ochre or other iron-rich earth pigments, where manganese content deepens the color to a darker, more brownish red. As in other samples, the zinc and calcium are most likely associated with the ground or preparation layer.

### Microstructural analysis and compositional validation by SEM–EDX

**Fig. 8:**
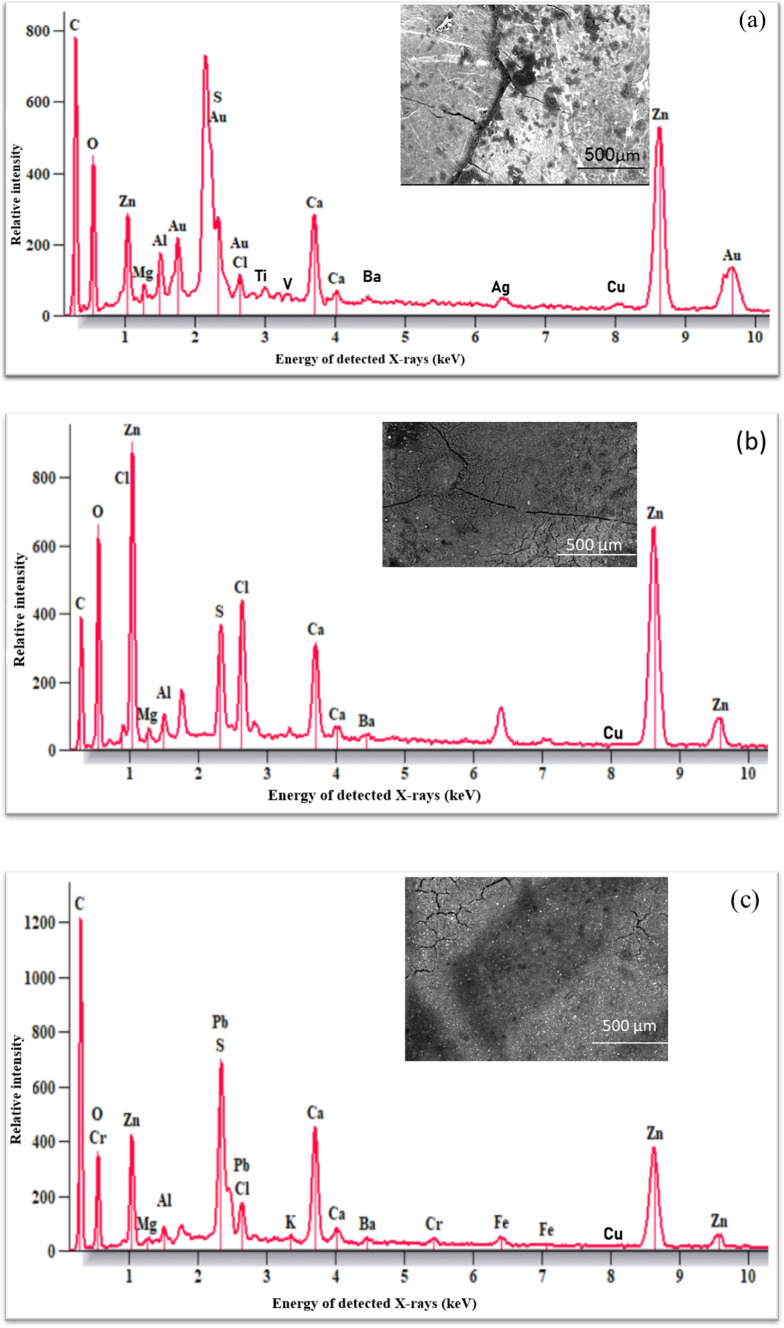

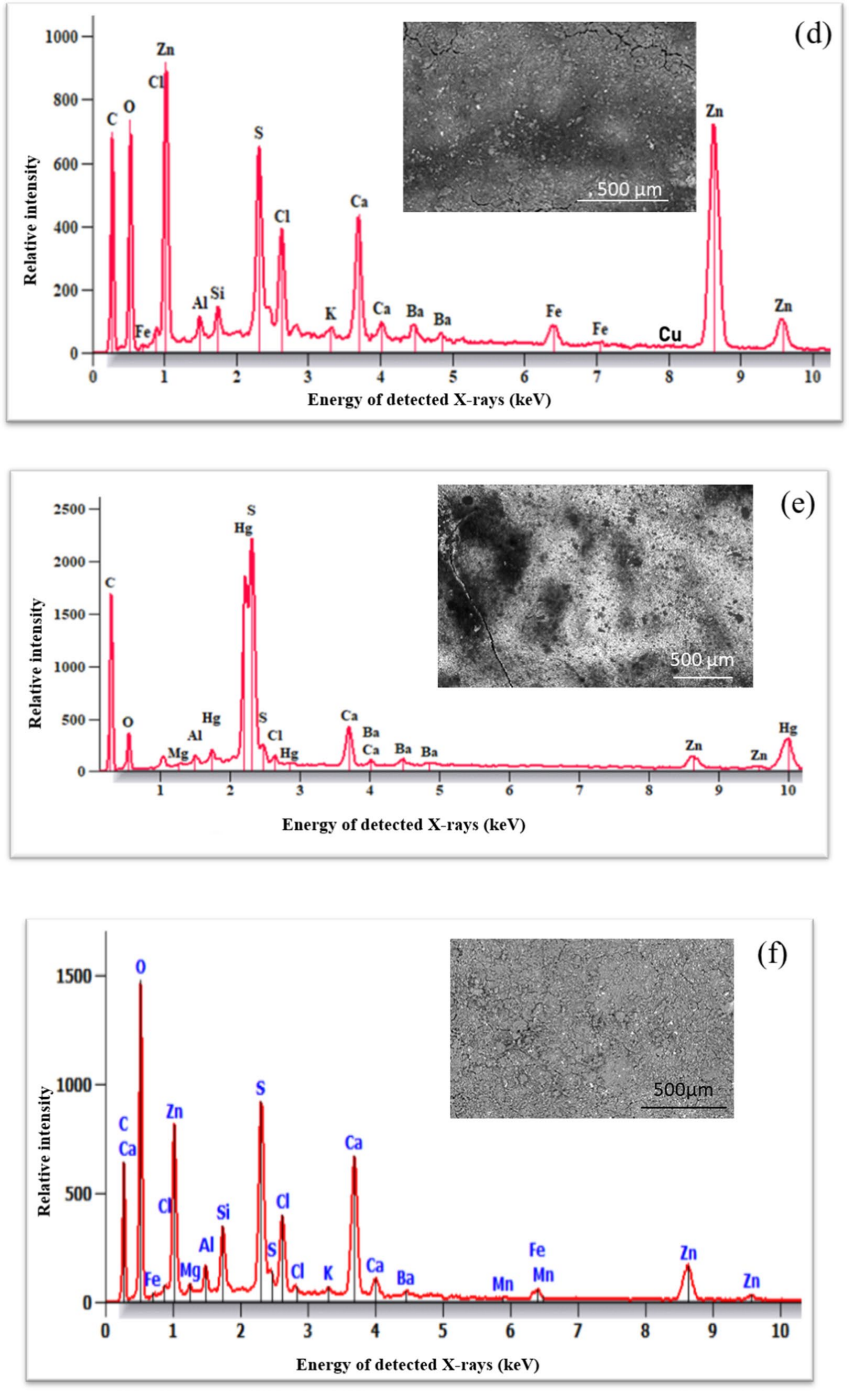
(**a**–**f**) EDX spectra and corresponding SEM micrographs (magnification 500×) of the analyzed samples: (**a**) Golden, (**b**) Brown, (**c**) Light green, (**d**) Blue, (**e**) Bright red, and (**f**) Dark red.

Scanning Electron Microscopy coupled with Energy-Dispersive X-ray spectroscopy (SEM–EDX) was employed to investigate microstructural morphology and obtain semi-quantitative elemental data to validate the LIBS results. SEM micrographs (insets in Figs. 8a–f) reveal heterogeneous surfaces with microcracks and pigment grains of varying sizes and shapes, features that are consistent with aged, hand-ground paint layers.

The EDX analyses provide crucial quantitative data for interpreting the LIBS spectra. In the golden sample (Fig. 8a), EDX reveals a gold layer with Au at 22.3 wt%, thereby confirming the LIBS-based identification of gilding. The brown sample (Fig. 8b) exhibits a very high zinc content (55.5 wt%) and Mn and Fe signals, suggesting a mixture in which zinc oxide (ZnO) serves as a primary white base, tinted with brown iron–manganese oxides. For the light green sample (Fig. 8c), EDX quantifies Zn (21.0 wt%), Ca (5.8 wt%), and Fe (1.0 wt%). The relatively low concentrations of chromophoric elements such as Cu and Cr, despite their detection by the more sensitive LIBS, indicate that the green hue may result from a low-concentration green pigment or a complex mixture, likely combined with a zinc-based white. In the blue sample (Fig. 8d), the EDX data show high Zn (35.0 wt%) and Ca (4.1 wt%), together with a lower Fe content (1.8 wt%). The measurable iron content (1.8 wt %), combined with the absence of copper in the EDX quantification (despite LIBS detection of Cu), suggests a minor contribution from iron-rich earth impurities or environmental dust rather than an independent iron-based mineral phase. Both LIBS and FTIR confirm that the primary chromophore of the blue paint layer is a copper-based compound (azurite), mixed with a barite base or extender.

The bright red sample (Fig. 8e) shows mercury (5.3 wt%) and sulfur (6.9 wt%), providing strong quantitative evidence for cinnabar (HgS). The co-occurrence of Zn (1.3–6.4 wt%) and Ca (2.6–2.9 wt%) supports the presence of a calcium- and zinc-carbonate-based ground or preparation layer. Finally, the dark red sample (Fig. 8f) contains Fe (2.1 wt%) and Mn (0.2 wt%) as the main chromophoric elements, consistent with an iron oxide pigment such as ochre or umber, while the substantial Ca (11.3 wt%) and Zn (20.8 wt%) again point to a preparation layer rich in calcium and zinc carbonates.

### Validation and integration of LIBS and EDX Data

**Fig. 9:**
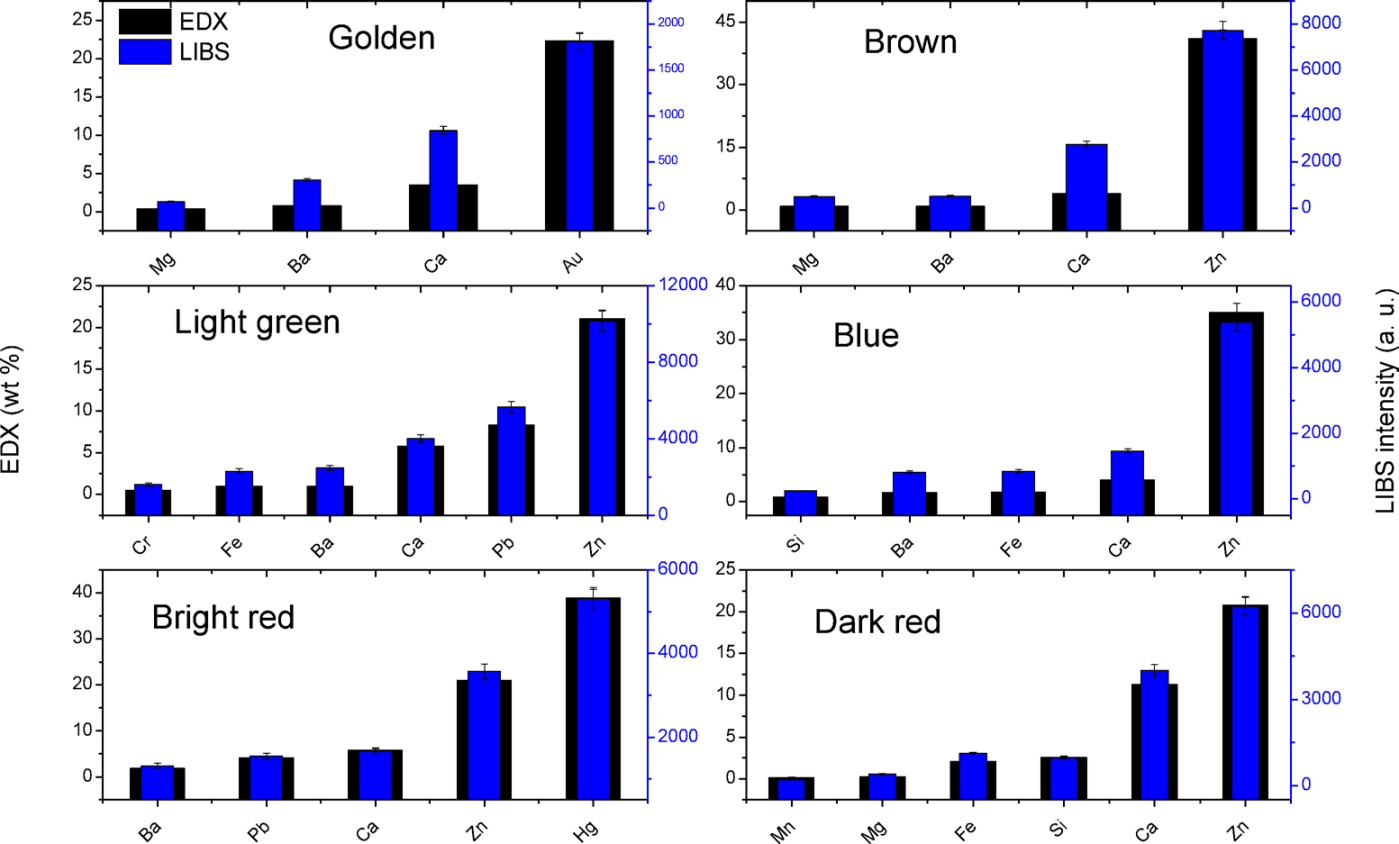
Validation of LIBS average spectral lines intensities results with EDX analysis.

A satisfactory correlation was observed between the qualitative/semi-quantitative LIBS data and the quantitative EDX results (Fig. 9). Elements with strong spectral line intensities in LIBS, such as Zn in the brown and blue samples and Au in the golden sample, correspond to high weight percentages in the EDX measurements. This cross-validation strengthens the reliability of the elemental assignments and demonstrates that LIBS, although not inherently quantitative without appropriate calibration, provides a robust and representative fingerprint of pigment composition. The combined application of LIBS and SEM–EDX therefore offers a powerful, complementary approach: LIBS enables rapid, wide-spectrum screening of virtually all elements, while SEM–EDX provides targeted quantitative validation and essential microstructural information. Together, they provide a more complete picture of the materials and techniques used in the wall paintings.

### Molecular analysis of pigments and binding media by FTIR

**Fig. 10:**
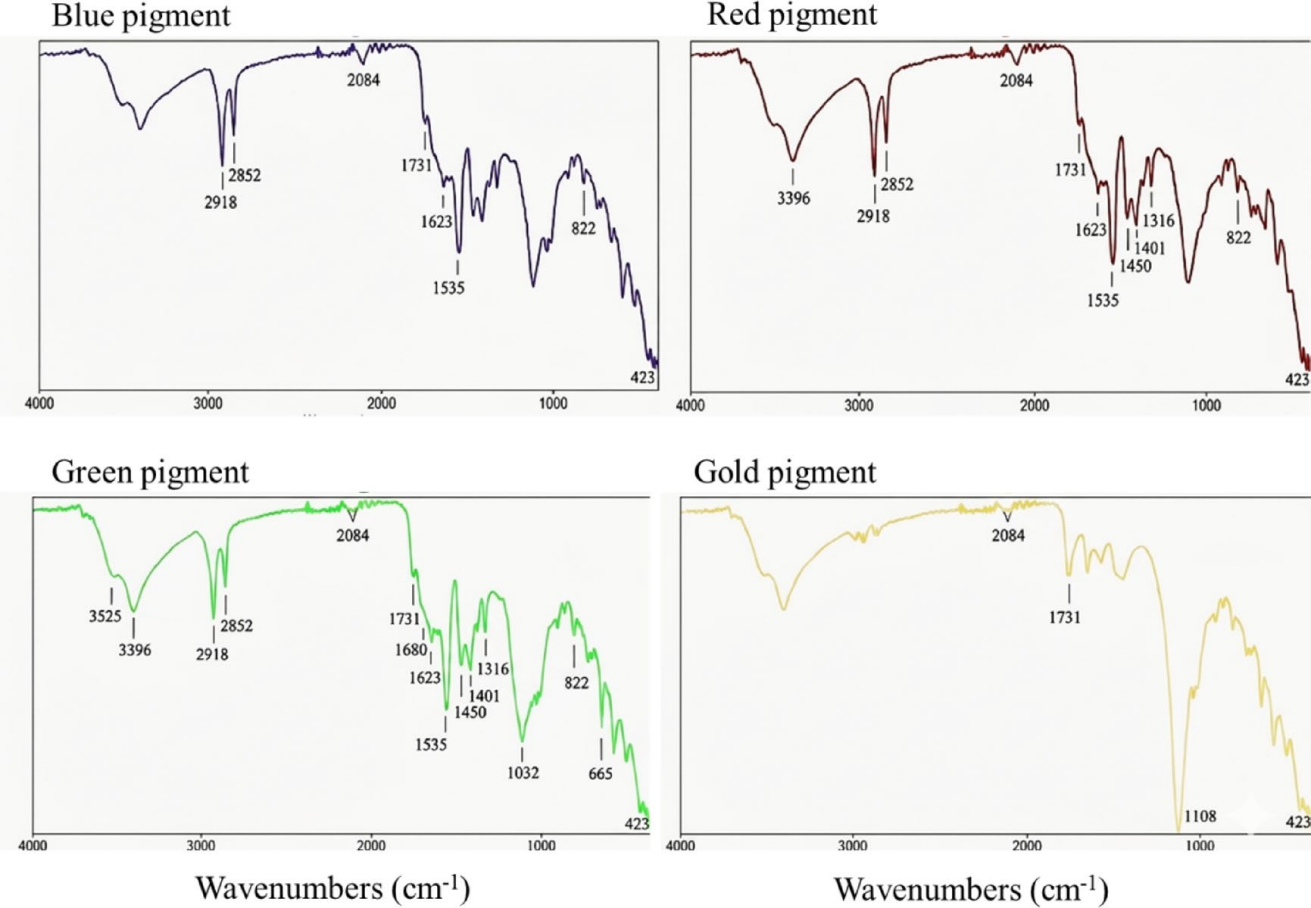
FTIR spectra of the Blue, Red, Green, and Gold paint samples, indicating binding media functional groups and mineral signatures.

Fourier Transform Infrared spectroscopy (FTIR) was used to identify molecular components, including both organic binding media and inorganic pigments. The FTIR spectra for the golden, green, red, and blue samples are shown in Fig. 10.

Regarding the binding media, spectral features consistent with an oil-based binder are present across all samples. The characteristic carbonyl ester (C=O) stretching band, typically observed between 1740 and 1750 cm^−1^ in fresh drying oils, appears in our samples at slightly lower wavenumbers (e.g., 1735, 1732, 1724 cm^−1^). This shift is a well-documented phenomenon in aged oil-paint films and is attributed to chemical interactions between the oil and the pigments, including the formation of metal soaps^25^. In addition, the aliphatic C–H stretching bands around 2918 and 2852 cm^−1^, together with the band at approximately 822 cm^−1^ attributed to linoxyn (a polymerization product of linseed oil), provide compelling evidence for the use of a drying oil, most likely linseed oil, as the binding medium^25^.

Regarding pigment identification, the FTIR spectrum confirms the green pigment as viridian, a hydrated chromium(III) oxide (Cr₂O₃·2H₂O). Cr–O supports this assignment vibrations at 532, 469, and 458 cm^−1^, the water bending mode at 1623 cm^−1^, and O–H stretching bands at 3525 and 3396 cm^−1^^26^. An absorption band at 665 cm^−1^ suggests the presence of sulfate, probably due to gypsum used as an extender or as an impurity. For the red pigment, the spectrum of the bright red sample matches that of red ochre. The characteristic Fe–O vibrations of hematite (α-Fe₂O₃) are observed at 423 cm^−1^^26^. Additional bands confirm the presence of associated minerals typical of earth pigments: kaolinite (Al–OH at 914 cm^−1^, Si–O–Al at 1108 cm^−1^), quartz (Si–O at 776 cm^−1^), and calcite (CaCO₃ at 1401, 873, and 721 cm^−1^)^27,28^.

The blue pigment spectrum is more complex. Bands associated with carbonate ions (approximately 1411 and 1541 cm^−1^), together with a weak feature at 923 cm^−1^ (merged OH bending), suggest the presence of a basic copper carbonate pigment such as azurite^29,30^. However, the very strong band at 1108 cm^−1^, along with bands at 665 and 599 cm^−1^, is a definitive indicator of sulfate ions (SO₄^2^⁻), pointing to the presence of barite (barium sulfate)^30^. This implies that the blue paint layer may be a mixture of azurite and barite, or that barite forms part of the underlying ground layer. The O–H stretching bands at 3396 and 3525 cm^−1^ may be associated with the azurite structure itself or with other hydrated mineral phases. The functional groups identified by FTIR for the green, red, and blue pigments are listed in Table 1S. FTIR data for the golden sample primarily characterize the organic binding medium, or mordant, rather than the gold itself, as metallic foils reflect infrared light and lack characteristic molecular infrared absorption bands.

## Conclusion

This study employed a multi-analytical spectrochemical approach to characterize the wall paintings of the early twentieth century Al-Qazdughli Palace in Cairo, generating essential information to support their conservation. The combined application of LIBS, SEM–EDX, FTIR, and optical microscopy proved to be a powerful and complementary methodology, enabling a comprehensive understanding of both the inorganic pigments and the organic binding media.

Elemental analysis by LIBS, confirmed by SEM–EDX, revealed distinct compositional fingerprints for each pigment. Gold layer was identified in the gilded areas, cinnabar in the bright red passages, and iron-manganese oxides in the brown and dark red regions. The detection of chromium in the green pigment, confirmed by FTIR as viridian, together with the complex mixture proposed for the blue pigment (azurite associated with barite), points to the concurrent use of traditional artists’ materials and newer industrial pigments available at the time. A particularly significant outcome is the consistent identification of an oil-based binding medium, most likely linseed oil, across all samples. This finding not only sheds light on the technological practices of the period but is also critical for designing appropriate cleaning, consolidation, and retouching strategies.

The strong agreement between the rapid, in situ–capable LIBS measurements and the quantitative microanalytical data from SEM–EDX highlights the potential of LIBS as a quasi-nondestructive screening tool for cultural heritage objects. Beyond deepening our understanding of the material culture and artistic techniques of early twentieth century Egypt, this research establishes a robust scientific foundation for the ongoing restoration of Al-Qazdughli Palace. The analytical data presented here will directly inform conservators’ choices of compatible materials and intervention methods, helping to ensure the long-term preservation of this historically and artistically significant monument for future generations.

## Supplementary Information

Below is the link to the electronic supplementary material.

Supplementary Material 1

## Data Availability

Data are available upon reasonable request from the authors.
