# Supplementary material for: Applying LIBS, SEM/EDX, and FTIR spectroscopic analysis for the conservation of cairene architectural heritage
Source: Sci Rep. 2026 Jun 9;16:17867. doi: 10.1038/s41598-026-56422-8 (PMC13250164; doi:10.1038/s41598-026-56422-8)
Supplement: Supplementary file 1 — Supplementary Material 1 [file 41598_2026_56422_MOESM1_ESM.docx]

**Table 1S. FTIR spectroscopic band assignments for binding media and pigments.**

| **Sample / Layer Component** | **Observed Wavenumber (cm^−1^)** | **Vibrational Mode & Functional Group Assignment** | **Assigned Material / Phase** |
| --- | --- | --- | --- |
| **All Samples (Binding Media)** | 1735, 1732, 1724 | C=O ester stretching (shifted due to aging/metal soaps) | Aged drying oil (Linseed oil) |
|  | 2918, 2852 | Aliphatic C-H stretching | Drying oil network |
|  | ~822 | Polymerization product vibration | Linoxyn |
| **Light Green Sample** | 532, 469, 458 | Cr-O metal-oxygen stretching | Viridian Cr_2_ O_3_ 2H_2_O |
|  | 1623 | H - O - H bending (water of hydration) | Hydrated chromium oxide |
|  | 3525, 3396 | O - H stretching modes | Hydrated matrix components |
|  | 665 | SO_4_^2-^ bending mode | Gypsum extender/impurity |
| **Bright Red Sample** | 423 | Fe - O lattice vibrations | Hematite (Fe_2_ O_3_) from Red Ochre |
|  | 914, 1108 | Al-OH bending & Si-O- Al stretching | Kaolinite (an associated earth mineral) |
|  | 776 | Si-O stretching | Quartz impurity |
|  | 1401, 873, 721 | Carbonate (CO_~~3~~_^2-^) stretching and bending modes | Calcite (CaCO_3_) ground |
| **Blue Sample** | 1411, 1541 | Carbonate (CO_3_^2-^) stretching modes | Azurite (Cu_3_(CO_3_)^2^(OH)_2_) |
|  | 923 | O-H bending (hydroxyl groups) | Azurite mineral phase |
|  | 1108 | SO_4_^2-^ asymmetric stretching | Barite (BaSO_4_) ground/extender |
|  | 665, 599 | SO_4_^2-^ bending modes | Barite component |
|  | 3396, 3525 | O - H stretching modes | Azurite / hydrated mineral matrix |
